# Serotonin Transporter and Plasma Membrane Monoamine Transporter Are Necessary for the Antidepressant-Like Effects of Ketamine in Mice

**DOI:** 10.3390/ijms21207581

**Published:** 2020-10-14

**Authors:** Melodi A. Bowman, Melissa Vitela, Kyra M. Clarke, Wouter Koek, Lynette C. Daws

**Affiliations:** 1 Department of Cellular and Integrative Physiology at University of Texas Health, San Antonio, TX 78229, USA; bowmanma@livemail.uthscsa.edu (M.A.B.); vitela@uthscsa.edu (M.V.); kyraclarke@mac.com (K.M.C.); 2 Department of Pharmacology at University of Texas Health, San Antonio, TX 78229, USA; koek@uthscsa.edu; 3 Department of Psychiatry at University of Texas Health, San Antonio, TX 78229, USA

**Keywords:** serotonin transporter, plasma membrane monoamine transporter, ketamine, isoflurane, serotonin clearance, antidepressant-like activity, chronoamperometry, tail suspension test, forced swim test

## Abstract

Major depressive disorder is typically treated with selective serotonin reuptake inhibitors (SSRIs), however, SSRIs take approximately six weeks to produce therapeutic effects, if any. Not surprisingly, there has been great interest in findings that low doses of ketamine, a non-competitive N-methyl-D-aspartate (NMDA) receptor antagonist, produce rapid and long-lasting antidepressant effects. Preclinical studies show that the antidepressant-like effects of ketamine are dependent upon availability of serotonin, and that ketamine increases extracellular serotonin, yet the mechanism by which this occurs is unknown. Here we examined the role of the high-affinity, low-capacity serotonin transporter (SERT), and the plasma membrane monoamine transporter (PMAT), a low-affinity, high-capacity transporter for serotonin, as mechanisms contributing to ketamine’s ability to increase extracellular serotonin and produce antidepressant-like effects. Using high-speed chronoamperometry to measure real-time clearance of serotonin from CA3 region of hippocampus in vivo, we found ketamine robustly inhibited serotonin clearance in wild-type mice, an effect that was lost in mice constitutively lacking SERT or PMAT. As expected, in wild-type mice, ketamine produced antidepressant-like effects in the forced swim test. Mapping onto our neurochemical findings, the antidepressant-like effects of ketamine were lost in mice lacking SERT or PMAT. Future research is needed to understand how constitutive loss of either SERT or PMAT, and compensation that occurs in other systems, is sufficient to void ketamine of its ability to inhibit serotonin clearance and produce antidepressant-like effects. Taken together with existing literature, a critical role for serotonin, and its inhibition of uptake via SERT and PMAT, cannot be ruled out as important contributing factors to ketamine’s antidepressant mechanism of action. Combined with what is already known about ketamine’s action at NMDA receptors, these studies help lead the way to the development of drugs that lack ketamine’s abuse potential but have superior efficacy in treating depression.

## 1. Introduction

Suicide rates have increased dramatically in recent years [1], and treatment resistant depression is a leading cause of suicide [2]. Treatment resistant depression affects up to 30% of people with depression, and, as its name implies, cannot be treated with traditional antidepressant medications such as selective serotonin reuptake inhibitors (SSRIs) [3], underscoring a dire need for novel medications that are effective in treating *all* individuals suffering from depression. Great interest has been generated from clinical studies showing that a single dose of ketamine produces rapid, dramatic, and sustained depression symptom relief, even in individuals suffering from treatment resistant depression [4,5,6]. Recently, the Federal Drug Administration approved (S)-Ketamine use exclusively in patients with treatment resistant depression [7]. However, the mechanism(s) by which ketamine exerts its pronounced and consistent antidepressant effects are not yet fully understood. Ketamine is a noncompetitive N-methyl-D-aspartate (NMDA) receptor antagonist. Not surprisingly, most preclinical studies have therefore focused on how blockade of NMDA receptors can exert antidepressant-like effects [8]. Ketamine triggers intracellular signaling pathways, including eukaryotic elongation factor 2 (eEF2) kinase, resulting in rapid increases in brain derived neurotrophic factor (BDNF) protein translation [8,9]. This increase in BDNF activates the mammalian target of rapamycin (mTOR) pathway, which in turn induces rapid synaptogenesis. Currently this is viewed as the primary mechanism underlying ketamine’s antidepressant-like effects in preclinical studies [8,10]. However, growing attention is turning to ketamine’s ability to increase extracellular serotonin.

The antidepressant effect of SSRIs is initiated by blockade of the serotonin transporter (SERT) and ensuing increase in extracellular serotonin [11,12]. Like SSRIs, ketamine also causes robust increases in extracellular serotonin in brain regions important in the pathophysiology of mood disorders [13,14,15]. Underscoring the importance of serotonin in ketamine’s antidepressant action, recent studies showed that ketamine was without antidepressant-like activity in rodents treated with parachlorophenylalanine (PCPA) to deplete serotonin [16,17,18,19]. However it is unclear whether ketamine’s ability to increase extracellular serotonin is mediated through glutamatergic control of serotonin release and/or via direct actions at SERT [20], the high-affinity, low-capacity (i.e., “uptake-1”) transporter for serotonin.

In addition to SERT, our lab, and others, found that low-affinity, high-capacity (i.e., “uptake-2”) transporters play a prominent role in regulating serotonin transmission [21,22,23,24,25]. Examples of “uptake-2” transporters include the plasma membrane monoamine transporter (PMAT) and organic cation transporters (OCTs). PMAT and OCTs are inhibited by decynium-22 (D22) and are collectively known as D22-sensitive transporters. Since PMAT is the most efficient transporter of serotonin among D22-sensitive “uptake-2” transporters, and is widely expressed in human brain, including limbic regions important in controlling mood [25], the possibility is raised that inhibition of PMAT-mediated serotonin uptake may also contribute to ketamine’s antidepressant actions. Therefore, we hypothesized that SERT and/or PMAT may contribute to the antidepressant-like effects of ketamine.

To explore this possibility, we used in vivo high-speed chronoamperometry to measure ketamine’s local effects on clearance of exogenously applied serotonin in CA3 region of hippocampus, a brain region important in regulating mood and for the actions of SSRIs [26], in wild-type and constitutive SERT knockout (−/−) and PMAT−/− mice. In parallel, we determined how ketamine’s effects on serotonin clearance mapped onto behavior in the tail suspension test (TST) and forced swim test (FST), commonly used behavioral assays that detect drugs with antidepressant potential [27,28]. To our knowledge, this is the first study to directly explore SERT and PMAT involvement in ketamine’s mechanism of action in vivo, and the first study to examine serotonin clearance in constitutive PMAT−/− mice. We also examined the effects of isoflurane anesthesia on serotonin release and clearance, prior to using this anesthetic for these in vivo high-speed chronoamperometry studies.

## 2. Results

### 2.1. Isoflurane Does Not Evoke Serotonin Release or Inhibit Serotonin Clearance at Concentrations Needed to Maintain Anesthesia

In the past, we have routinely used a mixture of alpha-chloralose and urethane to anesthetize rodents for chronoamperometric recordings. This was the anesthetic of choice because it reportedly does not interfere with monoamine transporters [29,30]. However, isoflurane has advantages over alpha-chloralose urethane, primary among them the ability to control the surgical plane of anesthesia more precisely and consistently. Isoflurane does not impact the dopamine transporter (DAT) [31] but has been reported to impact serotonin release in vitro and in vivo [32,33,34,35], although results are inconsistent. Therefore, before using isoflurane in our experiments, we confirmed that at concentrations needed to maintain anesthesia, isoflurane does not cause serotonin release or impact serotonin clearance in CA3 region of hippocampus. To this end, we carried out a concentration-response study using in vivo high-speed chronoamperometry in C57BL/6 male mice.

**Figure 1:**
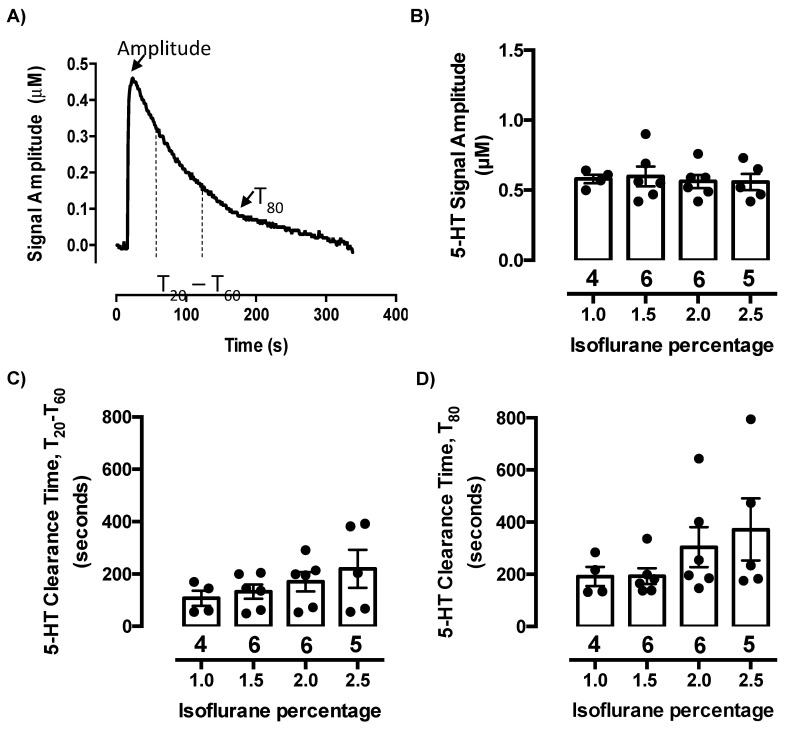
Isoflurane does not impact serotonin clearance at low concentrations. (**A**) Representative trace with signal parameters defined; (**B**) Serotonin signal amplitude did not change as a function of isoflurane percentage; (**C**) Serotonin clearance time (T_20_-T_60_) and (**D**) (T_80_) in seconds at varying concentrations of isoflurane (1.0–2.5%). There was no statistically significant difference in serotonin clearance time among the isoflurane concentrations. Circles represent individual mice. Data are mean ± S.E.M.

We found no evidence of measurable endogenous serotonin release following administration of any of the isoflurane concentrations, as evidenced by the baseline signal remaining stable (data not shown). Clearance of exogenously applied serotonin from extracellular fluid of the CA3 region of hippocampus was analyzed after each concentration of isoflurane ranging from 1.0–3.0% and given in 0.5% increments in a randomized order among mice. Peak signal amplitude (µM), time to clear between 20% and 60% (T_20_-T_60_, in s) of peak signal amplitude, and time to clear 80% (T_80_, in s) of peak signal amplitude were analyzed (Figure 1A). Serotonin was pressure-ejected into CA3 region of hippocampus to achieve signals of approximately 0.5 µM (Figure 1B). The amount (in pmol) of serotonin required to achieve signals of this amplitude did not vary as a function of isoflurane concentration and averaged 1.8 ± 0.3 pmol (one-way ANOVA: F(3,17) = 0.24, *p* = 0.89). We found that clearance times for serotonin were not different from those we have previously recorded using alpha-chloralose urethane anesthesia, with T_20_-T_60_ (Figure 1C) and T_80_ (Figure 1D) clearance values for signals of ~0.5 µM serotonin most commonly ranging from 100–200 s and 200–300 s, respectively. One-way ANOVA of T_20_-T_60_ and T_80_ serotonin clearance times revealed no significant difference among isoflurane concentrations (T_20_-T_60_: F(3,17) = 1.08, *p* = 0.38; T_80_: F(3,17) = 1.34, *p* = 0.29) (Figure 1C,D), with the highest concentration (2.5%) trending to increase serotonin clearance time, i.e., to inhibit serotonin clearance. Concentrations of isoflurane higher than 2.5% were not examined due to mice not surviving at 3.0% isoflurane. During our experiments, we maintained mice at an anesthetic plane using 1.0–1.5% isoflurane, as higher concentrations tend to interfere with respiration. Thus, these studies confirm that isoflurane, at doses required for maintenance of a surgical plane of anesthesia, do not evoke detectable serotonin release or interfere with serotonin clearance in vivo.

### 2.2. Comparison of Serotonin Clearance among SERT and PMAT Genotypes

**Figure 2:**
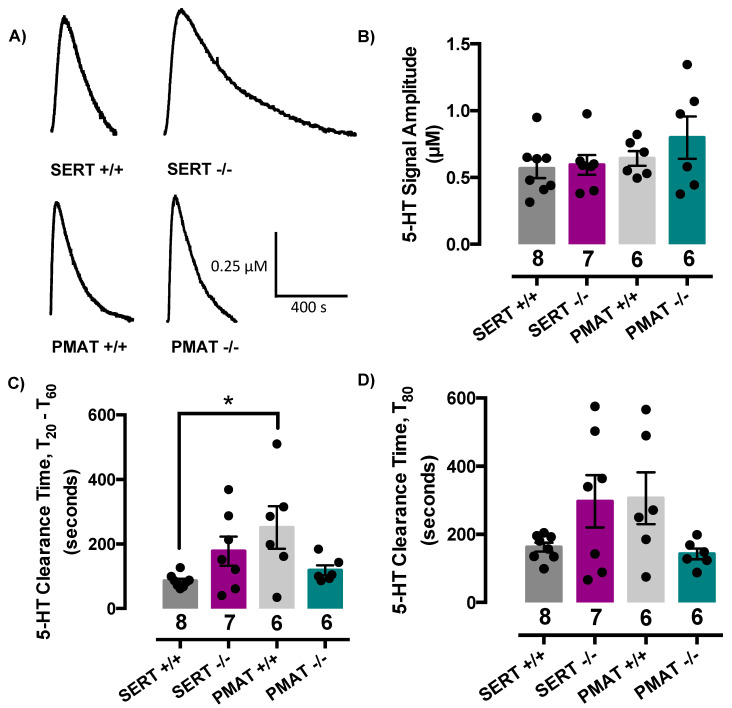
Comparison of serotonin clearance among serotonin transporter (SERT) and plasma membrane monoamine transporter (PMAT) genotypes. (**A**) Representative traces of basal serotonin clearance in SERT+/+, SERT−/−, PMAT+/+, and PMAT−/− mice. (**B**) Basal serotonin signal amplitude, and (**D**) T_80_ clearance time did not differ significantly among genotypes. There was an effect of genotype in basal T_20_-T_60_ serotonin clearance time (**C**) where PMAT+/+ cleared serotonin more slowly than SERT+/+ mice (but see Figure 3). Welch’s one-way ANOVA with Tukey’s post-hoc comparisons. * *p* < 0.05. Circles represent individual mice. Data are mean ± S.E.M.

We found no difference among genotypes in the pmol amount of serotonin required to achieve signal amplitudes of ~0.5 µM (2.0 ± 0.3, 1.8 ± 0.2, 1.8 ± 0.2 and 1.6 ± 0.1 pmol for SERT+/+, SERT−/−, PMAT+/+ and PMAT−/− mice, respectively). Representative serotonin signals for each genotype are shown in Figure 2A. Since a significant difference in variance for each of the serotonin signal parameters was detected among genotypes, we applied a Welch’s one-way ANOVA to the analyses of these data, which does not assume that all groups were sampled from populations with equal variances. Welch’s one-way ANOVA revealed that signal amplitudes did not differ among genotypes (F(3,11.8) = 0.56, *p* = 0.65) (Figure 2B), however there was a significant effect of genotype for T_20_-T_60_ serotonin clearance time (F(3,10.1) = 3.81, *p* = 0.05) but not for T_80_ clearance time (F(3,11.0) = 2.4, *p* = 0.12) (Figure 2D). Tukey’s post-hoc comparisons showed this difference in T_20_-T_60_ to be driven by PMAT+/+ mice clearing serotonin more slowly than SERT+/+ mice (*p* = 0.03) (Figure 2C). Given both lines are bred on a C57BL/6 background this finding was unexpected. That PMAT−/− mice clear serotonin as efficiently as SERT+/+ mice (Figure 2C,D) suggests that there is no consequence of constitutive PMAT knockout for serotonin clearance, at least at the concentration of serotonin used in these studies, and that this apparent difference between SERT+/+ and PMAT+/+ mice is peculiar to this cohort of PMAT+/+ mice. It is important to emphasize that the genotypes of all mice were double-checked to rule out the possibility that PMAT genotypes had been recorded inaccurately. We found this not to be the case, with all genotypes being confirmed as correct.

**Figure 3:**
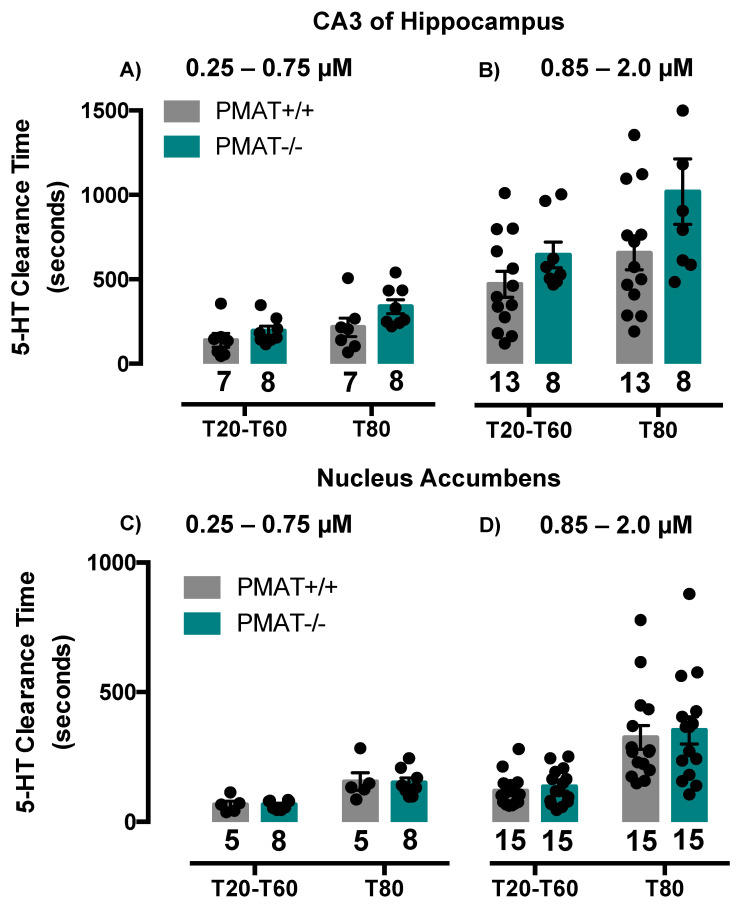
Serotonin clearance time (T_20_-T_60_ and T_80_) does not differ between PMAT+/+ and PMAT−/− mice, regardless of serotonin concentration. (**A**) There was no statistically significant difference between PMAT+/+ and PMAT−/− mice in serotonin clearance time (T_20_-T_60_ and T_80_) in CA3 of hippocampus at “low” concentration of serotonin (0.25 µM to 0.75 µM) or (**B**) “high” concentrations of serotonin (0.85 µM to 2.0 µM). (**C**) There was no statistically significant difference between PMAT+/+ and PMAT−/− mice in serotonin clearance time (T_20_-T_60_ and T_80_) in nucleus accumbens at “low” concentration of serotonin (0.25 µM to 0.75 µM) and (**D**) “high” concentrations of serotonin (0.85 µM to 2.0 µM). Note that the ordinates differ between CA3 region of hippocampus (panels A and B), and NAcc (panels C and D). Circles represent individual mice. Data are as mean ± S.E.M.

To investigate this apparent paradox further, we conducted additional experiments in two separate cohorts of PMAT+/+ and PMAT−/− mice and varied the concentration of serotonin delivered. In the first cohort, we measured serotonin clearance in the same brain region, CA3 region of hippocampus. In this cohort of PMAT+/+ mice, both T_20_-T_60_ and T_80_ clearance times were consistent with those of SERT+/+ mice for signals of similar amplitude and were ~100 s and 200 s, respectively (compare Figure 3A with Figure 2C,D). Figure 3A shows that for the lower serotonin concentrations tested (0.25 to 0.75 µM) there was no difference in clearance time from extracellular fluid of CA3 region of hippocampus for either T_20_-T_60_ (t(13) = 1.18, *p* = 0.26) or T_80_ (t(13) = 1.82, *p* = 0.09), between PMAT+/+ and PMAT−/− mice. As expected, at higher serotonin concentrations (0.85 to 2.0 µM), T_20_-T_60_ and T_80_ clearance times were longer than those of lower serotonin concentrations, and in PMAT+/+ mice were ~500 s and 650 s, respectively (compare grey bars in Figure 3A,B). Similar to the results obtained with low serotonin concentrations (0.25 to 0.75 µM), at higher serotonin concentrations (0.85 to 2.0 µM) there was no difference in serotonin clearance time for either T_20_-T_60_ (t(19) = 1.50, *p* = 0.15) or T_80_ (t(19) = 1.84, *p* = 0.08) between PMAT+/+ and PMAT−/− mice, although in PMAT−/− mice clearance trended to be slower than in PMAT+/+ mice, consistent with loss of the low-affinity, high-capacity PMAT (Figure 3B).

Using a separate cohort of mice, we examined serotonin clearance in the nucleus accumbens (NAcc) in order to determine if the lack of difference in serotonin clearance time in CA3 region of hippocampus between PMAT+/+ and PMAT−/− mice generalized to other brain regions. We found that serotonin clearance in NAcc was faster than in CA3 region of hippocampus, with T_20_-T_60_ and T_80_ clearance times of ~50 s and 150 s, respectively, for lower serotonin concentrations (0.25 to 0.75 µM), and ~100 s and 350 s, respectively, for higher serotonin concentrations (0.85 to 2.0 µM) (Figure 3C,D). As in hippocampus, there was no difference in serotonin clearance time between genotypes at either low (0.25 to 0.75 µM) serotonin concentrations (T_20_-T_60_: (t(11) = 0.02, *p* = 0.98) or T_80_: (t(11) = 0.11, *p* = 0.91)) or higher (0.85 to 2.0 µM) serotonin concentrations (T_20_-T_60_: (t(28) = 0.70, *p* = 0.49) or T_80_: (t(28) = 0.39, *p* = 0.70)) (Figure 3C,D). Based on findings from these more extensive investigations of serotonin clearance in PMAT+/+ and PMAT−/− mice, together with confirmation of mouse genotypes, we conclude that constitutive knockout of PMAT does not significantly impact serotonin clearance at the concentrations of serotonin tested. Moreover, we therefore conclude that the longer serotonin clearance in PMAT+/+ mice, relative to SERT+/+ mice reported in Figure 2, is exclusively due to sampling variation.

Consistent with our previous findings [21,36,37], serotonin clearance trended to be slower in SERT−/− mice than their wild-type counterparts (T_20_-T_60_: (t(13) = 2.15, *p* = 0.05); T_80_: (t(13) = 1.85, *p* = 0.09).

### 2.3. Ketamine Inhibits Serotonin Clearance in Wild-Type Mice, but Not in SERT−/− or PMAT−/− Mice

**Figure 4:**
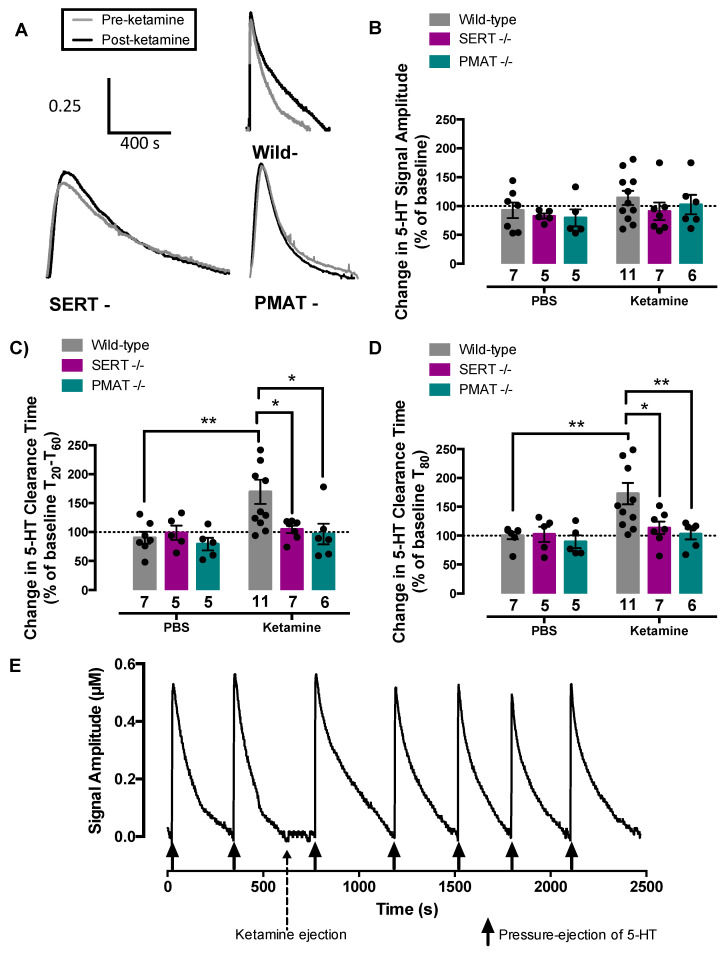
Ketamine significantly inhibited serotonin clearance in wild-type mice, but not in mice lacking SERT or PMAT. (**A**) Representative oxidation currents produced by pressure-ejecting serotonin into CA3 region of hippocampus before (grey) and after (black) local application of ketamine. Traces are superimposed for ease of comparison. (**B**) Percent change in serotonin signal amplitude did not differ between treatments and across genotypes. Percent change in serotonin clearance time (**C**) T_20_-T_60_, and (**D**) T_80_ increased in wild-type mice, but was unchanged in SERT−/− and PMAT−/− mice. (**E**) Representative time course for serotonin clearance in a wild-type mouse following ketamine ejection. Note that pressure-ejection of ketamine did not perturb baseline oxidation current, indicating that ketamine did not evoke detectable release of endogenous serotonin. * *p* < 0.05 ** *p* < 0.01. Circles represent individual mice. Data are mean ± S.E.M.

To test the hypothesis that ketamine increases extracellular serotonin by inhibiting its clearance via SERT and/or PMAT, we used in vivo high-speed chronoamperometry to measure clearance of exogenous serotonin from the extracellular fluid of CA3 region of hippocampus of anesthetized mice before and after local application of ketamine or phosphate buffered saline (PBS) vehicle in SERT+/+, PMAT+/+, SERT−/−, and PMAT−/− mice (Figure 4). Representative traces for serotonin clearance after ketamine in each genotype are shown in Figure 4A. Ketamine did not influence signal amplitude in any genotype (F(2,35) = 0.79, *p* = 0.46) (Figure 4A,B, Table 1), but significantly increased serotonin clearance time in both SERT+/+ and PMAT+/+ mice. As there was no statistically significant difference in the percent increase in serotonin clearance time following ketamine between SERT+/+ (T_20_-T_60_: 83 ± 38%; T_80_: 92 ± 25%) and PMAT+/+ (T_20_-T_60_: 59 ± 24%; T_80_: 58 ± 24%) mice [T_20-60_, t(9) = 0.55, *p* = 0.60; T_80_, t(10) = 0.97, *p* = 0.36], wild-type percent change data were pooled. Two-way ANOVA of the percent change in T_20_-T_60_ serotonin clearance time from pre-treatment (PBS vs. ketamine) values revealed a main effect of ketamine to prolong serotonin clearance time (main effect of treatment: (F(1,35) = 5.65, *p* = 0.02), main effect of genotype: (F(2,35) = 3.27, *p* = 0.05)), and interaction (F(2,35) = 2.88, *p* = 0.07) (Figure 4C). As per recommendations by Hsu et al. [38] and Maxwell et al. [39], post-hoc analyses were conducted even though the interaction was not statistically significant at the 5% level. PBS had no effect on serotonin clearance (in any genotype), whereas ketamine prolonged T_20_-T_60_ serotonin clearance in wild-type mice (*p* = 0.01), but not in SERT−/− (*p* = 0.98) or PMAT−/− mice (*p* = 0.99), as also evidenced by post-hoc comparisons of the effects of ketamine among genotypes (wild-type vs. SERT−/−, *p* = 0.05; wild-type vs. PMAT−/−, *p* = 0.03).

Similarly, two-way ANOVA of the percent change in T_80_ serotonin clearance time from pre-treatment values revealed a main effect of ketamine to increase serotonin clearance time (F(1,35) = 6.54, *p* = 0.02), main effect of genotype (F(2,35) = 4.01, *p* = 0.03), and interaction (F(2,35) = 2.92, *p* = 0.07) (Figure 4D). Post-hoc analysis showed PBS had no effect on serotonin clearance (in any genotype), whereas ketamine prolonged T_80_ serotonin clearance in wild-type mice (*p* = 0.01), but not in SERT−/− (= 0.98) or PMAT−/− (*p* > 0.99) mice, as also evidenced by post-hoc comparisons of the effects of ketamine among genotypes (wild-type vs. SERT−/−, *p* = 0.05; wild-type vs. PMAT−/−, *p* = 0.02). The effect of intrahippocampally applied ketamine to inhibit serotonin clearance persisted for approximately 20 min in both SERT+/+ and PMAT+/+ mice, returning to pre-drug clearance times by 30 min (Figure 4E). Average pre- and 2-min post-treatment signal amplitude, T_20_-T_60_, and T_80_ values are reported in Table 1, where paired t-test analyses revealed similar statistically-significant outcomes.

Taken together, these results suggest that loss of either SERT or PMAT is sufficient to eliminate ketamine’s ability to inhibit serotonin clearance. To determine if inhibition of serotonin clearance via SERT and/or PMAT is necessary for the antidepressant-like effects of ketamine we turned to the TST and FST.

### 2.4. Antidepressant-Like Effects of Ketamine Are Lost in Mice Lacking SERT or PMAT

**Figure 5:**
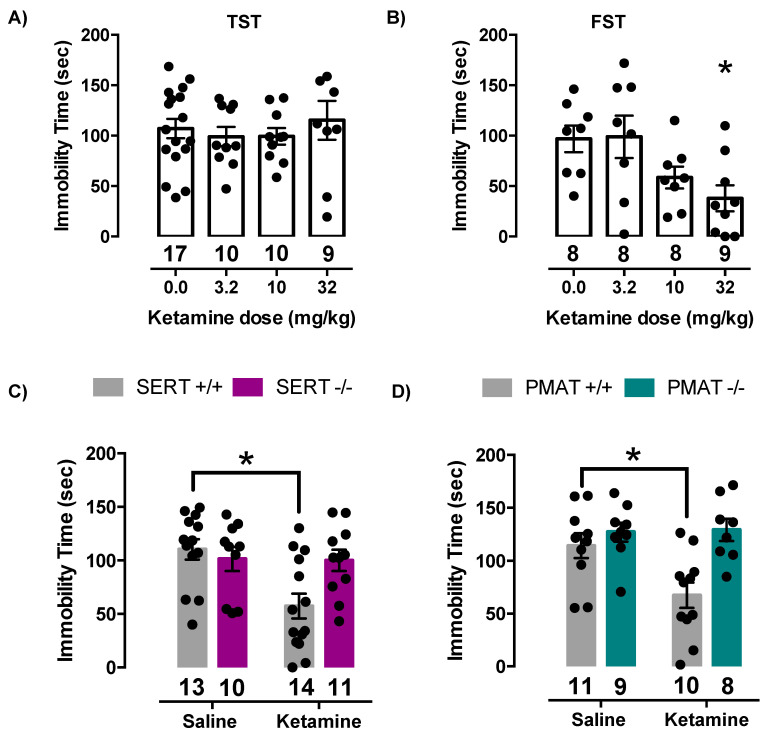
Ketamine lacks antidepressant-like effects in constitutive SERT or PMAT knockout mice. (**A**) Immobility time (s) in the tail suspension test and (**B**) Immobility time (s) in the forced swim test, in C57BL/6 mice given ketamine (3.2, 10, or 32 mg/kg) or saline. * *p* < 0.05 represents 32.0 mg/kg ketamine significantly different from saline. Tukey’s post-hoc test for multiple comparisons. (**C**) Immobility time in the FST after 32.0 mg/kg ketamine or saline administered one hour prior to testing in SERT+/+ or SERT−/− mice. * *p* < 0.05 represents 32.0 mg/kg ketamine significantly different from saline; Tukey’s post-hoc test for multiple comparisons. (**D**) Immobility time in the FST after 32.0 mg/kg ketamine or saline administered one hour prior to testing in PMAT+/+ or PMAT−/− mice. * *p* < 0.05 represents 32.0 mg/kg ketamine significantly different from saline. Tukey’s post-hoc test for multiple comparisons. Circles represent individual mice. Data are mean ± S.E.M.

To test the hypothesis that SERT and/or PMAT are necessary for the antidepressant-like effect of ketamine, we measured immobility time in the TST and FST in SERT+/+, PMAT+/+, SERT−/− and PMAT−/− mice. However, first we used wild-type (C57BL/6) mice to confirm, in our hands, that 32 mg/kg ketamine produced robust (near maximal) antidepressant-like effects, as reported by others [16,40,41]. Since the TST is the most used test for antidepressant-like activity in mice, we used this test first. We found that none of the ketamine doses tested (3.2, 10, or 32 mg/kg) produced antidepressant-like effects in wild-type mice (F(3,42) = 0.36, *p* = 0.78) (Figure 5A). These findings agree with those of others [42].

We turned to the FST, which has been shown to provide more consistent results when examining the antidepressant-like effects of ketamine [43]. Consistent with this literature, we found that ketamine produced dose-dependent and significant antidepressant-like effects in wild-type mice (F(3,29) = 4.15, *p* = 0.02). Tukey’s post hoc multiple comparisons test showed that 32.0 mg/kg ketamine was statistically different from saline (*p* = 0.03) (Figure 5B) and produced an effect of similar magnitude to that reported in the literature [16,40,41]. Therefore, for all subsequent experiments, 32.0 mg/kg ketamine was used to assess the antidepressant-like effect of ketamine using the FST.

We also looked for climbing behavior, which is routinely recorded during the FST when using rats as it aids in determining if the antidepressant-like actions of a drug are more strongly influenced by serotonergic or noradrenergic and dopaminergic systems; drugs that act to increase serotonin increase swimming, whereas drugs that act to increase norepinephrine and/or dopamine increase climbing [44,45]. We found that climbing behavior was essentially non-existent in mice given ketamine, suggesting that effects of ketamine were unlikely mediated by increased norepinephrine or dopamine signaling.

Replicating our findings in wild-type C57BL/6 mice, ketamine produced a robust antidepressant-like effect in SERT+/+ mice, which was absent in SERT−/− mice. Two-way ANOVA of time spent immobile revealed a statistically significant ketamine by genotype interaction (F(1,44) = 5.72, *p* = 0.02) (main effect of ketamine: F(1,44) = 6.27, *p* = 0.02; main effect of genotype: F(1,44) = 2.42, *p* = 0.13). Post-hoc analysis showed that 32.0 mg/kg ketamine significantly decreased immobility time in SERT+/+ mice compared to saline treated SERT+/+ mice (*p* = 0.003) and compared to saline treated SERT−/− mice (*p* = 0.03), however there was no effect of ketamine in SERT−/− mice (*p* > 0.99) (Figure 5C).

Similarly, ketamine produced an antidepressant-like effect in PMAT+/+ mice, which was absent in PMAT−/− mice. Two-way ANOVA of time spent immobile revealed a statistically significant ketamine by genotype interaction (F(1,34) = 4.82, *p* = 0.04) (main effect of ketamine: (F(1,34) = 4.03, *p* = 0.05); main effect of genotype: (F(1,34) = 11.14, *p* = 0.002). Post-hoc analysis showed that 32.0 mg/kg ketamine significantly decreased immobility time in PMAT+/+ mice compared to saline treated PMAT+/+ mice (*p* = 0.02) and compared to saline treated PMAT−/− mice (*p* = 0.002), however there was no effect of ketamine in PMAT−/− mice (*p* = 0.99) (Figure 5D). Taken together, these results indicate that elimination of either SERT or PMAT voids ketamine of its antidepressant-like effects.

### 2.5. Ketamine Does Not Influence Locomotor Activity of SERT+/+, SERT−/−, PMAT+/+, or PMAT−/− Mice

**Figure 6:**
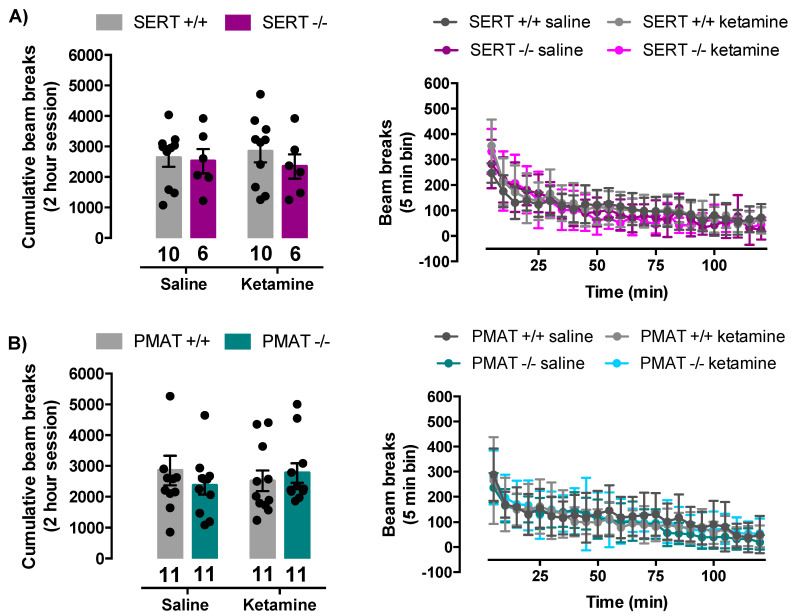
Ketamine did not significantly impact locomotor activity of SERT+/+, SERT−/−, PMAT+/+, or PMAT−/− mice. (**A**) There was no significant difference in locomotor activity of SERT+/+ and SERT−/−, or (**B**) PMAT+/+ and PMAT−/− mice, when given ketamine or saline. Bar graphs show total number of beam breaks in the two-hour recording period. Panels on the right show beam breaks in 5 min bins as a function of time. Circles represent individual mice. Data are mean ± S.E.M.

Locomotor activity of mice was not impacted by ketamine (32 mg/kg). In SERT+/+ and SERT−/− mice, there was no statistically significant interaction between ketamine treatment and genotype in locomotor activity of the mice (F(1,28) = 0.27, *p* = 0.61). There was also no main effect of ketamine treatment (F(1,28) = 0.003, *p* = 0.9) nor main effect of genotype (F(1,28) = 0.67, *p* = 0.42) (Figure 6A). Similarly, in PMAT+/+ and PMAT−/− mice, there was no statistically significant interaction between ketamine treatment and genotype (F(1,40) = 1.01, *p* = 0.32). There was also no main effect of ketamine treatment (F(1,40) = 0.008, *p* = 0.93) nor main effect of genotype (F(1,40) = 0.10, *p* = 0.75) (Figure 6B). Therefore, it is unlikely that effects of ketamine on locomotor activity affected outcomes in the FST.

## 3. Discussion

The key findings from this study are that ketamine inhibits serotonin clearance in the CA3 region of the hippocampus (Figure 4) and produces antidepressant-like effects (Figure 5) in a SERT- and PMAT-dependent manner. These data support an important role of serotonin in the antidepressant actions of ketamine through interaction with SERT and PMAT. Puzzling, however, is the finding that elimination of either SERT or PMAT was sufficient to render ketamine devoid of its serotonin clearance-inhibiting and antidepressant-like effects. Given that SERT−/− mice have a full complement of PMAT, and PMAT−/− mice have a full complement of SERT, it is surprising that ketamine’s ability to inhibit serotonin clearance and to produce antidepressant-like effects was lost, rather than diminished, suggesting a more complex interplay between ketamine and these transporters than previously thought. That these are constitutive knockouts likely comes into play, given that compensation in other systems is probable (discussed later). However, because SERT and PMAT overlap in their neuronal distribution, it is tempting to speculate that they may exist as heteromers, and only when in this configuration can ketamine exert inhibitory actions on serotonin clearance. Clearly, future research is needed to understand how constitutive loss of either SERT or PMAT, and compensation that occurs in other systems, is sufficient to abolish ketamine’s ability to inhibit serotonin clearance and produce antidepressant-like effects.

Although much research to date has focused on ketamine’s action as an antagonist at NMDA receptors, our findings add to increasing evidence showing a critical role for serotonin in the antidepressant effects of ketamine. The importance of serotonin is perhaps best exemplified by reports from several groups who found that the antidepressant-like effect of ketamine is lost when serotonin is depleted via PCPA treatment [16,17,18]. Consistent with an important role for serotonin in the antidepressant effects of ketamine, multiple studies have shown that ketamine increases extracellular serotonin, yet the mechanism(s) by which this occurs remains unclear [15,18,46,47,48,49,50]. Our studies point to ketamine-induced inhibition of serotonin clearance via SERT and PMAT as potential mechanisms.

The idea that ketamine blocks uptake of serotonin via SERT has been hypothesized since the 1970s. From that time to present day, investigators have used a variety of approaches to test this hypothesis, including measuring the ability of ketamine to inhibit uptake of tritiated ([^3^H])-serotonin into synaptosomes prepared from rodent brain [51], cell lines expressing murine or human SERT [52,53], and human platelets, which are rich in SERT [53]. Inhibition values (IC_50_ or Ki) range from ~50 to 230 µM, no doubt varying based on the system used to measure inhibition of [^3^H]-serotonin uptake. In 1982, Martin and colleagues [54] leveraged p-chloroamphetamine (PCA), a substrate for SERT that leads to depletion of serotonin, to gain insight into ketamine’s actions in vivo. They found that rats pre-treated with anesthetic doses of ketamine (80, 120, or 160 mg/kg), but not lower doses, were protected from the serotonin depleting effect of PCA, suggesting that ketamine blocks transport of PCA into serotonin neurons via SERT. While studies such as these have provided support for actions of ketamine at SERT, they suggest that inhibition of SERT by ketamine only occurs at concentrations much greater than those needed for antidepressant (or anesthetic) action. For example, in humans, plasma concentrations of ketamine needed for anesthesia are reported to be ~9 µM [55], while subanesthetic doses of ketamine used for treatment of depression result in plasma concentrations less than 1 µM [56,57,58]. Due to this, controversy has remained regarding the importance of blockade of serotonin uptake in the clinical efficacy of ketamine.

To gain a better understanding of the relationship between plasma and brain concentrations of ketamine and antidepressant-like effect in rodents, Can and colleagues [20] measured plasma and brain tissue concentrations of ketamine and its metabolites after systemic administration of 10 mg/kg ketamine in mice, a sub-anesthetic dose, which they found to have antidepressant-like effects [41]. Ketamine concentration peaked 10 min post injection at approximately 4 nmol/mL in plasma and 7 nmol/g in brain tissue, corresponding to approximately 4 µM and 7 µM, respectively. Using in vitro binding assays, they found that ketamine did not inhibit binding to SERT up to concentrations of 10 µM, and concluded that ketamine does not inhibit SERT at “therapeutically” relevant concentrations in mice [20]. These findings are in contrast to positron emission tomography (PET) studies in rhesus monkeys, where a subanesthetic dose of ketamine (1.5 mg/kg) was found to reduce binding of [^11^C]-3-amino-4-(2-dimethylaminomethyl-phenylsulfanyl)benzonitrile ([^11^C]DASB) to SERT [50]. More recently, Spies and colleagues [59] used PET imaging in humans to examine the effects of 0.5 mg/kg ketamine, a commonly used therapeutic dose in the treatment of depression. Though they did not find significant inhibition of [^11^C]DASB binding to SERT, they found a positive correlation between ketamine plasma levels and SERT occupancy, which trended to statistical significance. Since higher doses of ketamine are also used clinically, these studies encourage further investigation of ketamine’s action at SERT.

Our chronoamperometry studies are consistent with inhibition of serotonin uptake being an important mechanism of action of ketamine. Although we cannot know the precise concentration of ketamine reaching the recording electrode following pressure-ejection of drug locally into brain, we can estimate the concentration relatively accurately based on earlier studies (see Methods). We designed these studies so as to deliver approximately 2 µM in the area surrounding the recording electrode, a concentration less than the peak ketamine concentration (~7 µM) reported to reach brain in mice after a dose of 10 mg/kg [20]. Importantly, pressure-ejection of ketamine directly into brain has the advantage of allowing study of ketamine’s actions in vivo, in the absence of its metabolites. We found that in wild-type mice, ketamine robustly inhibited serotonin clearance in CA3 region of hippocampus, a brain region important for mood and therapeutic effects of antidepressants [26]. Consistent with an important role for SERT, this effect of ketamine was lost in mice constitutively lacking SERT. Moreover, this effect of ketamine was also lost in mice constitutively lacking PMAT. These data add support to findings that ketamine inhibits SERT at therapeutically relevant concentrations and brings a new player to the table: inhibition of PMAT.

The conclusion that ketamine inhibits serotonin uptake via SERT and PMAT at concentrations that produce antidepressant-like effects in mice is justified based on the clear-cut absence of this effect in SERT−/− and PMAT−/− mice. However, these data raise several questions, the most obvious being, why is elimination of either SERT or PMAT sufficient to render ketamine devoid of its serotonin clearance-inhibiting action? Given that both are constitutive knockouts, compensation in other systems (including other transporters or regulatory mechanisms) is likely at play. For example, it is known that OCT3 expression (mRNA and protein) is upregulated in SERT−/− mice [21]. It is possible that inhibitory actions of ketamine on serotonin clearance via PMAT in SERT−/− mice are masked by accelerated serotonin uptake via OCT3. A similar argument could be applied to PMAT−/− mice. To our knowledge, this is the first study to measure serotonin clearance in hippocampus and NAcc of PMAT−/− mice in vivo. To date, the only study investigating compensation in other transporters in PMAT−/− mice found no increase in mRNA expression of SERT, NET, DAT, OCT1, OCT2, and OCT3 in choroid plexus [60], however, protein levels were not measured and the possibility of functional upregulation of one or more of these transporters in PMAT−/− mice cannot be ruled out. In terms of functional studies, [^3^H]serotonin uptake into in vitro preparations of choroid plexus showed slower uptake in PMAT−/− mice compared to PMAT+/+ mice [60], however, it is difficult to ascertain the physiological relevance since the concentration(s) of substrate were not reported. Regardless, our in vivo findings suggest that at the serotonin concentrations studied here, serotonin clearance is not different between PMAT+/+ and PMAT−/− mice, indicating that compensatory mechanisms for serotonin clearance might be at play in constitutive PMAT−/− mice. This idea is further supported by PMAT−/− mice displaying normal behavior in a battery of tests, with only subtle differences in anxiety-like and coping behaviors compared to PMAT+/+ mice reported [61].

Importantly, our findings on the effects of ketamine to inhibit serotonin clearance correlated with the ability of ketamine to produce antidepressant-like effects. Ketamine produced robust antidepressant-like effects in wild-type mice, which were lost in SERT−/− and PMAT−/− mice. Antidepressant-like effects of ketamine in mice are consistently reported, yet the conditions under which this is detected varies. Unlike Can et al. [20] we did not find antidepressant-like effects of 10 mg/kg in either the TST or FST. However, this is in keeping with findings of others who have also found this dose to be ineffective in these tests [16,42,62,63]. Although there are some reports of ketamine producing antidepressant-like effects in mice in the TST [62,64], our data support those of others who find the TST insensitive to detecting antidepressant-like effects of ketamine [42]. Importantly, we found that ketamine dose-dependently reduced immobility time in the FST to a magnitude similar to reports of others [16,42,62,63], and this effect was clearly lost in SERT−/− and PMAT−/− mice.

Of course, because ketamine was delivered systemically in these behavioral studies, a role for metabolites of ketamine cannot be ruled out [20,41,46,49]. For example, Zanos and colleagues [41] found that (*2R*,*6R*)-hydroxynorketamine (HNK) has antidepressant-like effects similar to those of ketamine, and others found HNK to increase extracellular serotonin similar to ketamine [46,49]. We therefore cannot rule out the possibility that behavioral results from our study are due, at least in part, to HNK. However, data generated from chronoamperometry experiments, where ketamine was locally applied to CA3 region of hippocampus, suggest that the behavioral effects of ketamine were strongly driven by the parent compound, and not by its metabolites. That said, it will be important for future studies to interrogate the role of ketamine’s metabolites in inhibition of serotonin clearance in vivo.

While the antidepressant actions of ketamine have been the focus of this study, our results raise the possibility that SERT and/or PMAT might also be mechanisms involved in the addictive properties of ketamine, as well as its anesthetic actions. For example, it is well known that SERT is an important target for drugs of abuse, including psychostimulants such as 3, 4 methylenedioxymethamphetamine (MDMA, Ecstasy), and synthetic cathinones, known as bath salts, which inhibit transport of serotonin and/or cause reverse transport of serotonin via SERT. Ketamine at anesthetic doses is reported to increase serotonin, which may be involved in the psychotic-like symptoms occurring during ketamine emergence [58]. Along these lines, there are numerous other downsides to ketamine, including the possibility of hallucinations and dissociative effects, hypertension, tachycardia, and respiratory depression [58]. Whether SERT and/or PMAT are involved in these actions of ketamine remains unknown. Clearly, the mechanisms contributing to ketamine’s effects are complex, given its well-known action at NMDA receptors, its myriad effects on monoamine neurotransmission, and the recently reported dependency of ketamine on the opioid system for its antidepressant properties [65]. The avenues for future research dissecting these mechanisms of action are rich.

In sum, these studies provide evidence for an important role of SERT and PMAT in the serotonin clearance inhibiting and antidepressant actions of ketamine, and pave the way for future studies to understand the seemingly paradoxical loss of these effects in mice lacking either SERT or PMAT.

## 4. Materials and Methods

### 4.1. Animals

Naïve adult male SERT+/+, SERT−/−, PMAT+/+, and PMAT−/− mice bred on a C57BL/6 background, or C57BL/6 mice, from our in-house colonies were used for all experiments. Genotype comparisons were made among littermates, bred by +/− intercross to produce all three genotypes within a litter. Mice are backcrossed (+/+ × −/− to yield +/−) every 6^th^ generation. Sex differences in the antidepressant-like effect of ketamine have been previously reported, where female animals were found to be more sensitive [66,67,68]. For the present studies we selected a dose of ketamine (32 mg/kg) known to produce (near maximal) antidepressant-like effects in males, and used only male mice so as to reduce the number of animals needed for this initial study. However future studies in females will be necessary to determine the generality of present findings. We confirmed that this dose produced robust antidepressant-like effects in our hands by carrying out dose-response studies in wild-type (C57BL/6) mice before commencing studies to compare the antidepressant-like effects of ketamine in SERT+/+, SERT−/−, PMAT +/+, and PMAT −/− mice. Mice were between 3 and 12 months of age for all experiments. Mice were housed in plastic cages (29 cm × 18 cm × 13 cm) containing 7090 Teklad sani-chip bedding (Envigo, East Millstone, NJ) and maintained on a 12/12 hr light/dark cycle (lights on at 7:00 am) in a temperature-controlled (24 °C) vivarium. Mice were weaned at postnatal day 21 and housed with same sex littermates with no more than 5 mice per cage. Mice were given free access to food (Teklad LM-485 mouse/rat sterilizable diet 7012 chow (Envigo, East Millstone, NJ)) and water. All procedures were conducted in accordance with the National Institute of Health Guide for the Care and Use of Laboratory Animals (Institute of Laboratory Animal, Resources, Commission of Life Sciences, National Research Council, https://grants.nih.gov/grants/olaw/Guide-for-the-Care-and-use-of-laboratory-animals.pdf), and with the approval of the Institutional Animal Care and Use Committee, The University of Texas Health Science Center at San Antonio (protocol number: 20020014AR; Originally approved in 2002, current expiration 30 Sept. 2021).

### 4.2. High-Speed Chronoamperometry

In vivo high-speed chronoamperometry was used to examine transporter efficiency by recording real-time serotonin clearance. Experiments were conducted using methods adapted from Daws and Toney [21,24,69,70]. Carbon fiber electrodes were fabricated based on methods from Gerhardt [71,72] and described in Daws and Toney, and Williams et al. [70,73]. In brief, a single carbon fiber (30 µm diameter) was sealed in fused silica tubing (Schott North America, Elmsford, NY, USA). Nafion coating (5% solution; Sigma-Aldrich, St. Louis, MO, USA) was applied to carbon fiber electrodes to prevent anions in extracellular fluid from coming in contact with the carbon fiber [69,70]. Sensitivity to serotonin and its metabolite 5-hydroxyindoleacetic acid (5-HIAA) were measured by calibrating electrodes to increasing concentrations of serotonin (0.2 to 1.0 µM in 0.2 µM increments) in the presence of 5-HIAA (250 µM). Only those electrodes with a selectivity ratio for serotonin over 5-HIAA greater than 100:1 and a linear response (r^2^ ≥ 0.9) to serotonin were used.

The Nafion-coated carbon fiber electrode was attached to a four-barrel glass micropipette (FHC, Bowdoin, ME, USA) with their tips separated by 200 µm. Barrels of the micropipette were filled with either serotonin (200 µm), ketamine (400 µm), or phosphate-buffered saline (PBS). Note that the concentration of neurotransmitter and drug reaching the recording electrode is estimated to be ~200-fold less than the barrel concentration, based on our routine findings that pressure-ejection of ~20 nL of 200 µM serotonin, 200 µm away from the recording electrode, results in signal amplitudes of ~0.5–1.0 µM [69,70]. Thus, in these experiments, the concentration of ketamine reaching the electrode is estimated to be ~2 µM.

The electrode assembly was lowered into either the CA3 region of the hippocampus (anteroposterior −1.93 and mediolateral +2.0 from bregma; −2.0 from dura; [74]) or nucleus accumbens (anteroposterior +1.36 and mediolateral +1.85 from bregma; −5.0 from dura at 10° angle from midline; [74]) of an anesthetized mouse. Isoflurane (5%) was used to initially anesthetize the mouse and 1.0–1.5% isoflurane was used throughout the experiment to maintain anesthesia. Body temperature was maintained at 36–37 °C by a water circulated heating pad.

FAST-16 system (Quanteon, Nicholasville, KY, USA) was used for the high-speed chronoamperometric recordings. Oxidation potentials consisted of 100 ms pulses of +0.55 V alternated with 900 ms intervals during which the resting potential was maintained at 0 V. The active electrode voltage was applied with respect to a silver chloride reference electrode placed in the contralateral superficial cortex. Oxidation and reduction currents were digitally integrated during the last 80 ms of each 100 ms voltage pulse.

Exogenous serotonin was pressure ejected into the CA3 region of the hippocampus. Once reproducible serotonin signals (~0.5 µM, ~2 pmol in 15 nL) were obtained, ketamine (54 pmol in 136 nl) or an equivalent volume of PBS was pressure ejected locally into the CA3 region of the hippocampus. Following ketamine or PBS pressure ejection, serotonin was pressure ejected every 5 min until serotonin clearance time returned to pre-drug values. The T_80_ time course (time it takes for the signal to decline by 80% of the peak signal amplitude), T_20_-T_60_ time course (time it takes for the signal to decline by 20–60% of the peak signal amplitude), and peak signal amplitude were analyzed (Figure 1A).

At the completion of the experiment, an electrolytic lesion was made to mark the placement of the electrode tip. Brains were removed, frozen, and stored at −80 °C for histological analysis. Brains were thawed to −18 °C and sliced into 20 µm thick sections and stained with thionin for verification of electrode placement. Two animals were eliminated from the analyses due to electrode placement outside of CA3 region of hippocampus.

### 4.3. Effects of Isoflurane on Serotonin Clearance

Male C57BL/6 mice were placed into a Plexiglas chamber (25 cm × 10 cm × 10 cm) and 5% isoflurane was applied to the chamber via a precision vaporizer (Protech International Inc., TX, USA). Once completely anesthetized, mice were moved to the stereotaxic frame with their nose placed inside the anesthesia mask. During surgery isoflurane was maintained at 2%, but once the electrode was lowered into the brain isoflurane was set to 1.5% and subsequently maintained at 1.5% or 1.0% for approximately 30 min before the start of the experiment.

During the experiment, mice were administered varying concentrations of isoflurane in a randomized order to examine the effect of isoflurane on serotonin clearance. Each concentration of isoflurane (ranging from 1.0–3.0%, given in 0.5% increments) was administered for 5 min before pressure ejecting serotonin. Five minutes was chosen to allow adequate time for the effects of the new concentration to occur, and to detect any release of endogenous serotonin that may be elicited by isoflurane.

### 4.4. Tail Suspension Test

Tail suspension test (TST) experiments were performed as described by Steru et al. [75,76]. Mice received either saline, 3.2, 10.0, or 32.0 mg/kg ketamine intraperitoneally (i.p.) one hour prior to testing. These doses of ketamine were chosen based on previous studies examining the antidepressant-like effect of ketamine in mice in the TST [77]. Before testing, an aluminum bar (2 cm x 0.3 cm x 10 cm) was fastened to the tail of the mouse using adhesive tape and was placed at a 90° angle to the longitudinal axis of the tail with 3–4 cm between the base of the tail and the end of the bar. Opposite the tail taped end of the bar was a hole that was used to secure the bar to a hook in the top of a visually isolated box (40 cm × 40 cm × 40 cm). Mice were suspended for six minutes with the ventral surface and front and hind limbs of the mouse facing a digital video camera outside of the box. After the six-minute recording session ended, mice were removed from the bar and returned to their home cage. Moments of immobility were defined by the absence of initiated movement and included passive swaying. Total immobility was recorded by observers blinded to treatment conditions. A mouse was excluded from the study if it climbed its tail for 3 or more seconds. Seven mice were excluded on these grounds, with mice from all genotypes being involved. Mice were randomly assigned to treatment conditions and were tested only once.

### 4.5. Forced Swim Test

Forced swim test (FST) experiments were performed as described by Lucki [78]. A separate cohort of mice were used in the FST. Mice received either saline or 3.2, 10.0, or 32.0 mg/kg ketamine i.p. 60 min before testing. During testing, mice were confined in transparent cylindrical Plexiglas containers (19 cm diameter, 25.4 cm height, and 15 cm water level) containing water (23–25 °C). The test lasted for 6 min and the entire swim session was recorded. Once the test ended, mice were removed from the water and dried off with paper towels before being placed into a holding cage on a heating pad to aid in drying. Once dry, mice were returned to their home cages. Digital recording cameras were placed above the Plexiglas containers to record the mice from above. This vantage point was used in order to view all four limbs during the test. Moments of swimming and immobility were scored by an observer blinded to treatment. Immobility was defined as absence of active behaviors and remaining passively floating or making minor limb movements to stay afloat. Behaviors during the last four minutes of the swim session were scored [79].

### 4.6. Locomotor Activity

As ketamine has been shown to affect locomotor activity [80,81,82], it was important to assess whether ketamine impacted locomotor activity to rule out possible decreases in immobility time being due to ketamine-induced hyperactivity. We examined the effect of 32 mg/kg ketamine on locomotor activity as we found this to be the only dose to significantly reduce immobility time in the FST. None of the doses influenced immobility time in the TST. Locomotor boxes (30 cm × 15 cm × 15 cm), located within sound-attenuating ventilated chambers (MED Associates Inc., St. Albans, VT, USA), were equipped with infrared emitters and receivers (Multi-Varimex, Columbus Instruments, Columbus, OH, USA). Naïve mice received an i.p. injection of either saline or 32.0 mg/kg ketamine and immediately placed into a locomotor box. Locomotor activity of mice was examined for 2 h post injection with locomotion measured as infrared beam breaks per 5 min period.

### 4.7. Drugs

Ketamine hydrochloride (Sigma-Aldrich, St. Louis, MO, USA) was dissolved in physiologic saline and injected i.p. at doses expressed as salt weight per kilogram of body weight. The injection volume was 10 mL/kg.

### 4.8. Data Analysis

Data were analyzed using Prism 6.0 (GraphPad, San Diego, CA, USA). Data are expressed as mean ± S.E.M. *p* ≤ 0.05 was considered statistically significant for all analyses.

#### 4.8.1. High-Speed Chronoamperometry

For isoflurane studies, effects on pmol amount serotonin delivered to achieve signal amplitudes of ~0.5 µM and on serotonin clearance parameters (T_20_-T_60_ and T_80_ in seconds) in C57BL/6 mice were analyzed using a one-way ANOVA (isoflurane concentration), with Tukey’s post-hoc multiple comparisons. Amount of serotonin pressure-ejected, signal amplitude, and basal time course parameters among SERT and PMAT genotypes were analyzed using a Welch’s one-way ANOVA (genotype), as variances were found to differ significantly among groups. Tukey’s post-hoc comparisons were made when relevant. Changes in serotonin signal parameters (amplitude, T_20_-T_60_, and T_80_) induced by ketamine or PBS vehicle were expressed as a percent change from pre-drug/vehicle values, and analyzed using a two-way ANOVA (treatment x genotype) followed by Tukey’s post-hoc multiple comparisons test. Raw data for pre- and post-drug/vehicle values shown in Table 1 were analyzed by paired t-tests.

#### 4.8.2. Tail Suspension Test and Forced Swim Test

The dose-dependency of ketamine’s antidepressant-like effects was first examined in C57BL/6 mice using the TST and FST. Dose-response data were analyzed using a one-way ANOVA (dose) followed by Tukey’s post hoc multiple comparisons test. Since there were no significant effects of ketamine (at any dose) in the TST, the antidepressant-like effect of ketamine in SERT+/+, SERT−/−, PMAT+/+, and PMAT−/− mice was examined using the FST. The antidepressant-like effect of the highest dose of ketamine (32 mg/kg) was compared with saline across the different genotypes using a two-way ANOVA (treatment x genotype) followed by Tukey’s post hoc multiple comparisons test.

#### 4.8.3. Locomotor Activity

Locomotor activity was assessed following saline or 32 mg/kg ketamine injection across all genotypes of mice (SERT+/+, SERT−/−, PMAT+/+, and PMAT−/−) using two-way ANOVA (treatment x genotype) followed by Tukey’s multiple comparisons test.

## 5. Conclusions

At “therapeutically” relevant concentrations in mice, ketamine inhibited serotonin clearance and produced antidepressant-like effects in wild-type mice, but not in SERT−/− and PMAT−/− mice. Taken together with existing literature [15,16,17,18,19,45,47,48,49,50], a critical role for serotonin and its inhibition of uptake via SERT and PMAT cannot be ruled out as important contributing factors to ketamine’s antidepressant mechanism of action. These findings pave the way for future experiments to interrogate the role, and mechanism of action, of SERT and PMAT in the antidepressant actions of ketamine, which will add to literature suggesting that concurrent blockade of SERT and “uptake-2” transporters, such as PMAT, has greater antidepressant efficacy than SSRIs alone [21,23,24]. Combined with what is already known about ketamine’s action at NMDA receptors, these studies will help lead the way to development of drugs that lack ketamine’s abuse potential but have superior efficacy in treating depression.

## Figures and Tables

**Table 1:** Summary of pre- and post-serotonin signal amplitudes and time course (T_20_-T_60_ and T_80_) for serotonin clearance 2 min following pressure-ejection of phosphate buffered saline (PBS) or ketamine into CA3 region of hippocampus.

Genotype		SERT+/+	SERT+/+	SERT−/−	SERT−/−	PMAT+/+	PMAT+/+	PMAT−/−	PMAT−/−
Drug		PBS	Ketamine	PBS	Ketamine	PBS	Ketamine	PBS	Ketamine
N		4	5	5	7	3	6	5	6
Amp	Pre	0.44 ± 0.08	0.62 ± 0.09	0.46 ± 0.06	0.59 ± 0.07	0.56 ± 0.11	0.64 ± 0.06	0.74 ± 0.16	0.77 ± 0.15
(µM)	Post	0.47 ± 0.16	0.66 ± 0.18	0.37 ± 0.32	0.59 ± 0.19	0.48 ± 0.15	0.74 ± 0.08	0.62 ± 0.18	0.72 ± 0.11
T_20_-T_60_	Pre	109 ± 13	83 ± 6	173 ± 39	178 ± 45	361 ± 99	251 ± 66	138 ± 54	171 ± 51
(s)	Post	114 ± 22	149 ± 28 #	186 ± 56	188 ± 50	282 ± 102	326 ± 55 #	109 ± 46	171 ± 60
T_80_	Pre	194 ± 20	155 ± 18	273 ± 56	297 ± 77	475 ± 173	306 ± 76	183 ± 55	193 ± 49
(s)	Post	202 ± 19	278 ± 21 **	305 ± 86	321 ± 78	487 ± 221	422 ± 81 *	165 ± 60	206 ± 67

Pre- and post-drug values for peak signal amplitude and clearance time (T_20_-T_60_ and T_80_) 2 min after local application of PBS or ketamine. ** *p* < 0.01 * *p* < 0.05 significantly different from pre-drug value (paired t-test), # *p* = 0.08, Mean ± S.E.M.

## Abbreviations

+/+	Wild-type
−/−	Knockout
[^11^C]DASB	[^11^C]-3-amino-4-(2-dimethylaminomethyl-phenylsulfanyl)benzonitrile
5-HIAA	5-hydroxyindoleacetic acid
ANOVA	Analysis of variance
BDNF	Brain derived neurotrophic factor
DAT	Dopamine transporter
FST	Forced swim test
HNK	(*2R*,*6R*)-hydroxynorketamine
NMDA	*N*-methyl-d-aspartate
NAcc	Nucleus accumbens
NET	Norepinephrine transporter
OCT1	Organic cation transporter 1
OCT2	Organic cation transporter 2
OCT3	Organic cation transporter 3
PBS	Phosphate buffered saline
PCA	p-Chloroamphetamine
PCPA	Parachlorophenylalanine
PET	Positron emission tomography
PMAT	Plasma membrane monoamine transporter
SERT	Serotonin transporter
SSRI	Selective serotonin reuptake inhibitor
TST	Tail suspension test
